# Magnetic resonance imaging of sugar beet taproots in soil reveals growth reduction and morphological changes during foliar *Cercospora beticola* infestation

**DOI:** 10.1093/jxb/erv109

**Published:** 2015-04-01

**Authors:** Simone Schmittgen, Ralf Metzner, Dagmar Van Dusschoten, Marcus Jansen, Fabio Fiorani, Siegfried Jahnke, Uwe Rascher, Ulrich Schurr

**Affiliations:** Forschungszentrum Jülich GmbH, Institut für Bio-und Geowissenschaften, IBG-2: Plant Sciences, Wilhelm-Johnen-Straße, D-52425 Jülich, Germany

**Keywords:** *Beta vulgaris*, *Cercospora beticola*, *Cercospora* leaf spot, magnetic resonance imaging (MRI), morphological taproot changes, non-invasive phenotyping.

## Abstract

*Cercospora* leaf spot (CLS) infection can cause severe yield loss in sugar beet. Introduction of *Cercospora*-resistant varieties in breeding programmes is important for plant protection to reduce both fungicide applications and the risk of the development of fungal resistance. However, *in vivo* monitoring of the sugar-containing taproots at early stages of foliar symptoms and the characterization of the temporal development of disease progression has proven difficult. Non-invasive magnetic resonance imaging (MRI) measurements were conducted to quantify taproot development of genotypes with high (HS) and low (LS) levels of susceptibility after foliar *Cercospora* inoculation. Fourteen days post-inoculation (dpi) the ratio of infected leaf area was still low (~7%) in both the HS and LS genotypes. However, during this period, the volumetric growth of the taproot had already started to decrease. Additionally, inoculated plants showed a reduction of the increase in width of inner cambial rings while the width of outer rings increased slightly compared with non-inoculated plants. This response partly compensated for the reduced development of inner rings that had a vascular connection with *Cercospora*-inoculated leaves. Hence, alterations in taproot anatomical features such as volume and cambial ring development can be non-invasively detected already at 14 dpi, providing information on the early impact of the infection on whole-plant performance. All these findings show that MRI is a suitable tool to identify promising candidate parent lines with improved resistance to *Cercospora*, for example with comparatively lower taproot growth reduction at early stages of canopy infection, for future introduction into breeing programmes.

## Highlight

By employing magnetic resonance imaging to study below-ground sugar beet development, early changes in taproot growth and anatomy could be correlated with the first symptoms of *Cercospora* leaf spot disease.

## Introduction

*Cercospora* leaf spot (CLS), caused by infection by *Cercospora beticola*, is one of the most destructive foliar diseases of *Beta vulgaris* L. ssp. *vulgaris* grown for sugar production in temperate climate zones worldwide (Weiland and Koch, 2004). CLS causes up to 40% crop loss and can lead to complete yield loss in the absence of fungicide treatments (Shane and Teng, 1992; Rossi *et al.*, 2000). Disease management is achieved by a combination of crop rotation, fungicide application, and cultivation of resistant sugar beet cultivars (Shane and Teng, 1992). However, due to increasing fungal resistance to different classes of fungicides (Weiland and Koch, 2004) and the demand for integrated pest management, introduction of genetic resistance becomes of greater importance for the preventive control of CLS epidemics (Rossi, 1995). *Cercospora beticola* invades sugar beet leaves through the stomata and changes from biotrophic (non-symptomatic) to necrotrophic (tissue damaging) growth after producing the non-host toxins cercosporin and beticolin, a photosensitizer and a compound that changes membrane permeability by forming ion channels, respectively (Daub and Ehrenshaft, 2000; Goudet *et al.*, 2000). Symptoms start to develop with single leaf spots coalescing into extended lesions that eventually lead to the destruction of the whole photosynthetically active leaf area. Severely affected sugar beet plants generally respond by generating new leaves and thereby consume sugars reallocated from the taproot, which results in overall yield loss (Saito, 1966; Rossi *et al.*, 2000; Berger *et al.*, 2004; Holtschulte *et al.*, 2010). At high infection pressure, resistant sugar beet cultivars exhibit lower disease severity and display marginal foliar damage. However, under lower infection pressure, such resistant genotypes produce lower biomass and sugar yield compared with highly susceptible genotypes (Shane and Teng, 1992; Saito, 1966; Smith and Campbell, 1996; Rossi *et al.*, 1999). Wild relatives such as *Beta vulgaris* spp. *maritima* generally show lower susceptibility (Coons *et al.*, 1955) and nowadays serve as donor germplasm for introduction of CLS resistance in modern sugar beet breeding lines.

It is well known that leaf infestation reduces taproot growth and yield, yet there is still limited understanding of the morphological and anatomical responses of taproot development during extended vegetation periods (weeks to months). Anatomical traits of the sugar beet taproot might be indicative of biotic stress resistance. Sugar beet shows a specific secondary thickening of the taproot resulting from parallel cambial activity in several concentric rings, producing vascular and parenchymatic tissue (Artschwager, 1926; Zamski and Azenkot, 1981). Due to cell division and enlargement, each cambial ring increases in thickness, thus increasing beet diameter starting with the innermost cambial rings. Depending on the cultivar and developmental stage, taproots can have 12–15 cambial rings in which sugar is stored (Fowler *et al.*, 1999). During early stages of development of the taproot, one cambial ring can be induced and provided with photoassimilates from different leaves that have 3–10 vascular connections to the root system (Zamski and Azenkot, 1981).

Traditional approaches to observing root systems are, for example, destructive digging up, weighing, and scanning (Box and Ramsuer, 1993; Samson and Sinclair, 1994; Trebbi and McGrath, 2009). These methods do not allow the observation of the same specimen at different developmental stages. Such invasive studies showed reduction of harvested taproot biomass, decrease of sugar accumulation, and metabolic changes following the fungal infection (Smith and Campbell, 1996; Trebbi and McGrath, 2009; Weltmeier *et al.*, 2011). Detailed studies of the vascular system and taproot morphology are based on light microscopic and autoradiography approaches using harvested tissue (Artschwager, 1926, 1930; Zamski and Azenkot, 1981). Despite the high level of detail, these studies necessarily offer only snapshots of the morphological and physiological effects at the specific developmental stage selected for harvest. In contrast, analysing the *in vivo* effects on root development requires non-destructive methods. While progress has been made in phenotyping of the shoot with imaging sensors (Bock *et al.*, 2010; Fiorani and Schurr, 2013), comparatively few studies focused on below-ground measurements. Lately, magnetic resonance imaging (MRI) of plant organs (Borisjuk *et al.*, 2012), especially of soil-grown plants, has proved to be feasible for imaging roots in soil (Metzner *et al.*, 2014), indicating the potential of high-resolution 3D imaging to advance knowledge of root responses to environmental factors (Jahnke *et al.*, 2009; Rascher *et al.*, 2011; Jansen *et al.*, 2014a).

The aim of this study was to detect early effects of CLS on taproot growth and to analyse long-term effects on cambial ring structure alteration of two sugar beet genotypes, differing in resistance levels, by monitoring structural traits of individual plants with MRI. These morphological changes were indeed found already at 14 days post-inoculation (dpi), and a clear difference in growth performance between the genotypes from the very beginning was detected. Following fungal inoculation, the plants with low susceptibility were slightly more strongly affected in taproot growth than highly susceptible plants.

## Materials and methods

### Plant cultivation

To highlight differences in disease effects on plants with contrasting disease susceptibility to *C. beticola*, sugar beet inbreed lines of *B. vulgaris* ssp. *vulgaris* with different resistance levels to *C. beticola* were chosen which were characterized as having high (HS) and low (LS) susceptibility [identification nos 8RF5006 (HS) and 6S_1541 (LS), KWS SAAT AG, Einbeck, Germany]. Plants were grown under greenhouse conditions up to developmental stage 18 (experiment 1) and 16 (experiment 2) based on the BBCH scale (Hess *et al.*, 1997). Plants were grown in PVC tubes (81mm internal diameter and 40cm length) in a mixture of homogenized agricultural topsoil and coarse sand (1:2; v/v), as previously described by Metzner *et al.* (2014). Water (15ml per watering unit) and NPK nutrient supply (Hakaphos Blau, COMPO Expert GmbH, Münster, Germany) was adjusted stepwise to the BBCH development stage and applied with an automated watering system (GARDENA Manufacturing GmbH, Ulm, Germany). Nutrient concentration was increased from 0.01% (v/v) within the first 3 weeks (1–2 times per day), 0.03% in the next 4 weeks (3–4 times), 0.05% in the following week (four times), and 0.07% in the next 5 weeks (four times), and a final amount of 0.12% (three times) till harvest. The photoperiod was set to a 16h light/8h dark cycle with >200 μmol m^–2^ s^–1^ at leaf level by additional illumination in the greenhouse (Master SON-T Pia Agro 400W, Philips Deutschland GmbH, Hamburg, Germany). Greenhouse temperature was set to minimum 15 °C (STH9T Fan heater, Helios Ventilatoren, Villingen-Schwenningen, Germany). In experiment 1, plants were cultivated in the period from April to August. During this period, the air temperature in the greenhouse reached on average 24±2 °C (with a range between 17 °C and 47 °C). The maximum air temperature of 43–47 °C was detected in June (3 d) and in July (5 d). In experiment 2, plants were cultivated in the period from October to March. During this period, the air temperature reached on average 19±1 °C (with a range between 16 °C and 38 °C), and a maximum of 36–38 °C was detected on 2 d in October.

### Pathogen cultivation

The pathogen *C. beticola* (hemibiotrophic Ascomycete) was grown on 50% vegetable juice (Gemüsemix, Eckes-Granini Deutschland GmbH, Nieder-Olm, Germany) agar plates (1.5% agar, Sigma-Aldrich Chemie GmbH, Taufkirchen, Germany) at 60% humidity and 26 °C in a climate chamber for 3 weeks. To increase conidia production, plates were kept under UV light for 3 d. Conidia were carefully scraped from the plates with an object slide to prepare an aqueous suspension. The concentration was set to 3×10^4^ conidia ml^–1^ using a haemocytometer (Thoma chamber, Carl Roth GmbH + Co. KG, Karlsruhe, Germany), and 0.1% (v/v) Tween-20 (Sigma-Aldrich Chemie GmbH) was added.

### Inoculation

Plants were inoculated with a *C. beticola* conidial (isolate Herensen, KWS SAAT AG) suspension as described previously (Schmidt *et al.*, 2008) and kept under 80–100% relative humidity and temperature between 15 °C (night) and 35 °C (day) for 1 week. The light intensity was set to >300 μmol m^–2^ s^–1^ 24h after inoculation. Control plants were mock-inoculated with a 0.1% (v/v) Tween-20 solution. Inoculated plants were analysed in terms of foliar disease scoring and taproot growth in comparison with non-inoculated plants as described below. In experiment 1, inoculation of plants (*n*=5 plants per treatment) was conducted 71 d after planting (DAP) in April, and harvest took place on 160 DAP (or 89 dpi). Due to time restrictions in the availability of the MRI facility, the focus was on the highly susceptible genotype (*n*=4 plants per treatment) in experiment 2 to confirm the results of experiment 1. Plants were inoculated on 77 DAP in October, and harvested 175 DAP (or 98 dpi).

### Leaf measurements

Foliar disease severity was scored visually as the percentage of infected area per leaf. Since the scoring methods described to date do not cover the full range of possible leaf infection, two methods were combined: the lower range of 0–3% infected leaf area was classified with a modified scale based on Shane and Teng (1992), whereas the large scale of 3–100% was estimated according to the scale of Rossi *et al*. (1989). Mean values of disease severity were calculated by pooling all leaves of one genotype (all plants per treatment) and dividing by the number of plants. Leaves were included at the time point of the first visible symptoms. Disease severity was cumulated over time, so that leaves scored as having 100% infected leaf area were included in the leaf pool of subsequent observation time points. The number of diseased leaves per plant increased within dpi and was calculated as:

$$\begin{array}{l} \text{Average no}.\text{ of infected leaves per plant }\\ \left({\text{ILP}}_{\text{a}}\right)=\left(\text{no}.\text{ of infected leaves per genotype}\right)/\\ \left(\text{no}.\text{ of investigated plants per genotype}\right) \end{array}$$ (1)

Additionally, leaf area was measured manually with a ruler to compare leaf area between genotypes by multiplying the measured length and width of each leaf by the specific leaf area factor of 0.7014. To compute the leaf area factor (slope of linear correlation), several sheet templates, shaped like sugar beet leaves, were weighed and correlated with their measured quadratic area. The average leaf area per plant was calculated as the mean of five plants per treatment (*n*=5 ±SE).

### Taproot measurements

MRI is a well-known (3D, volumetric) imaging technique often used for medical imaging. General background information on the physics of the signal generation, acquisition, and processing is given in, for example, Haacke *et al.* (1999). As previously described (Metzner *et al.*, 2014), measurements were conducted with a vertical bore 4.7T magnet equipped with gradient coils providing 300 mT m^–1^ (Varian, Oxford, UK), that are required to make images. A spin echo multislice sequence was used with an echo time of 5.4ms and a repetition time of 2 s (sequence provided as part of the instrument package by Varian). Each measurement took 9min. Further relevant measurement parameters were: slice thickness of 1.5mm, 64 slices, a field of view (FOV) of 70×70mm^2^, and pixel size of 273×273 μm^2^. Plants were measured at 20 °C.

### Data analysis

Images were analysed with the software package MeVisLab (MeVis Medical Solutions AG, Bremen, Germany) in combination with Matlab (Mathworks, Ismaning, Germany) and the open source Matlab toolbox AEDES (version r172, aedes.uef.fi). To extract taproot signal from the background, signal had to be segmented from noise and unwanted sources of signal (e.g. water in soil pockets, petioles) by manually setting an intensity threshold minimizing background noise and maximizing the sample signal under visual control (Metzner *et al.*, 2014). The biomass was then calculated by multiplying the volume by the standard density of 1.17±0.16g ml^–1^ (mean ±SD), that was derived from sugar beets of the cultivar ‘Pauletta’ (KWS SAAT AG) by weighing the harvested beets (*n*=21 plants) directly after the MRI measurement (Metzner *et al.*, 2014).

Taproot parameters such as volume, length, width, and ring thickness were then analysed, and mean values (*n*=5 and *n*=4 plants ±SE) were calculated per plant treatment. The relative growth rate (RGR) of taproot volume (cm^3^) was calculated for the first 14 d after inoculation as

$$\text{RGR }\left(\%{\text{ d}}^{–\text{1}}\right)=\text{1}00\;\left[\text{ln }\left(V_{t}{}_{\text{2}}\;V_{t}{{}_{\text{1}}}^{–\text{1}}\right)\;{\left(t_{\text{2}}–t_{\text{1}}\right)}^{–\text{1}}\right]$$ (2)

where V_t1_ is the volume at day *t*_1_ and *V*_*t*2_ is the volume at day *t*_2_, as previously described (Walter and Schurr, 1999). The successive cambial rings were numbered based on their distance from the taproot central core, with ring 1 being the innermost cambial ring. Ring thickness was measured manually for each cambial ring with a software tool written in MeVisLab. Because the growth patterns of the inner four cambial rings and of all outer rings were similar, the width of inner and outer rings was summed. Significance tests were conducted to compare either treatments at one time point (*t*-test), over time [one-way analysis of variance (ANOVA)], or regarding treatment and time×genotype (two-way ANOVA) using the software SigmaPlot (Systat Software GmbH, Erkrath, Germany).

## Results

Two experiments were conducted with the aim of following disease effects on shoot and root development of sugar beets after foliar *Cercospora* inoculation. In experiment 1, two genotypes, a HS and an LS genotype, were compared to find differences in disease reactions due to their level of susceptibility. Experiment 2 focused on the HS genotype for observation of morphological changes of taproots during foliar disease progression.

### Disease effects on the shoot of genotypes with contrasting susceptibility

**Fig. 1.**
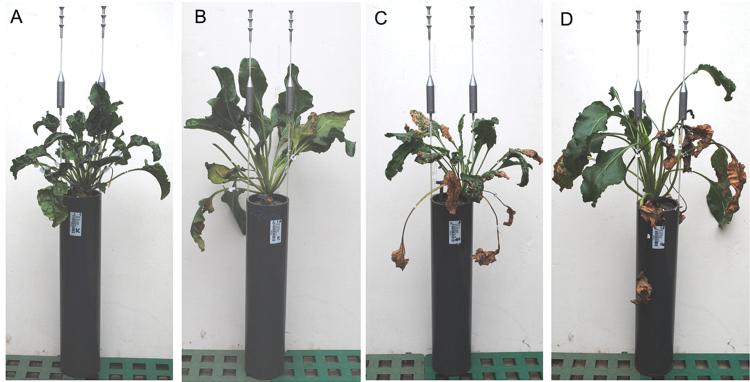
Images of HS and LS sugar beet plants, inoculated or not inoculated with *C. beticola*. Non-inoculated treatment of the HS (A) and LS (B) genotype, and inoculated treatment of the HS (C) and LS (D) genotype. For plant cultivation, plants were grown in PVC pots of 40cm depth to allow deeper root growth. Each pot was labelled with an individual code (white labels) to match measurements to plants. Images were acquired shortly before harvest at 90 dpi.

**Fig. 2.**
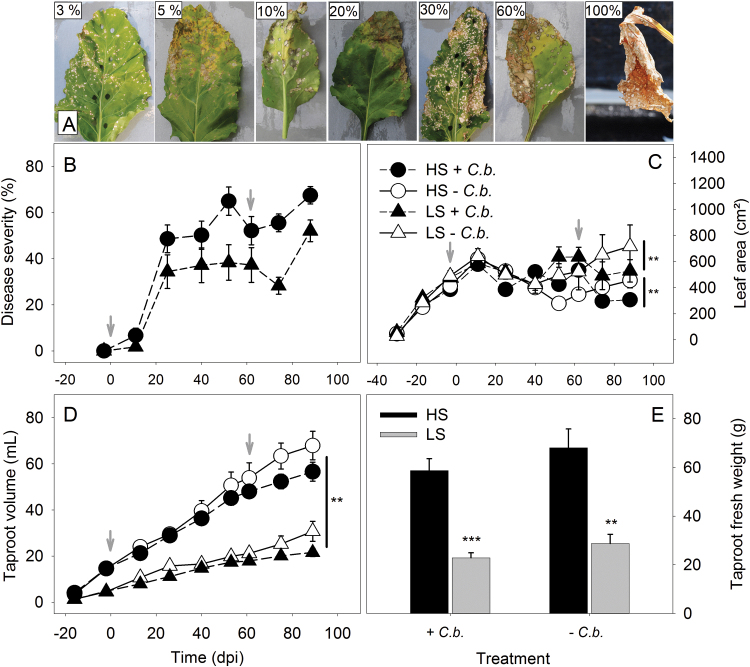
Shoot and root development of the HS and LS sugar beet plants. (A) Representative, visually scored leaf samples illustrating different levels of disease severity (% infected leaf area per leaf). Due to tissue sampling, two leaves (with 3% and 30% disease severity) showed punched holes. (B) Disease severity progression (mean ±SE) with increasing average number of infected leaves per plant (ILP_a_), 1–16 leaves for LS and 7–20 leaves for HS from 14 to 89 dpi. (C) Leaf area development during –28 to 89 dpi; asterisks indicate statistically significant differences between the inoculated (+ *C.b.*) and non-inoculated (– *C.b.*) treatment of the HS and LS genotype at 89 dpi analysed by a *t*-test, *P*<0.05. (D) Taproot volume measured non-invasively with MRI; asterisks indicate statistically significant differences between the treatments of the HS and LS plants analysed at 90 dpi with one-way ANOVA, *P*<0.01. The + *C.b.* and – *C.b.* treatments of each genotype did not differ significantly (*P*=0.159 between the treatments of LS and *P*=0.165 of HS). (E) Taproot fresh weight after harvest at 90 dpi. Comparing the HS and LS genotypes, asterisks indicate statistically significant differences between the + *C.b.* plants (*P*<0.001) and the – *C.b.* plants of the HS and LS genotype (*P*<0.01) analysed by a *t*-test. (C, D, E) The arithmetic mean of each plant was calculated during –14 dpi to 90 dpi (mean ±SE; *n*=5 per treatment). The two dates of inoculation with *C. beticola* are indicated by grey arrows.

In experiment 1, sugar beets grew in pots until harvest at 90 dpi, showing phenotypic differences between genotypes with respect to the development of non-inoculated (Fig. 1A, B) and inoculated shoots (Fig. 1C, D). After inoculation, CLS disease severity increased in leaves of both genotypes from single leaf spots to the final loss of the inoculated leaves (Fig. 2A). Disease severity was estimated by the combination of two scoring scales (Rossi *et al*. 1989; Shane and Teng, 1992), and the average number of infected leaves per plant (ILP_a_) was calculated (see Equation 1).

The HS plants showed stronger disease severity than the LS plants. The HS genotype showed a 7% necrotic leaf area (ILP_a_=7) at 11 dpi, increasing to 65% (ILP_a_=7) after the first inoculation at 52 dpi. A second inoculation was conducted to infect additional leaves, because disease symptoms did not spread from infected to non-infected leaves. Prior to an increased disease severity, freshly infected leaves reduced the average disease severity for both genotypes. During 62–8 dpi, an increase of leaf damage from 52% (ILP_a_=7) to 67% (ILP_a_=20) was scored for the HS genotype (Fig. 2B). During the same periods, the LS genotype showed an increase of foliar damage from 2% (ILP_a_=1) to 38% (ILP_a_=6) and the second inoculation caused an increase from 37% (ILP_a_=6) to 52% (ILP_a_=16).

In addition to disease severity, leaves were measured to analyse the loss of photosynthetically active leaf area. The comparison between the genotypes showed that the non-inoculated and the inoculated LS plants reached a higher leaf area than the HS plants. In both treatments and for each genotype, leaf area developed similarly until 14 dpi (Fig. 2C). During 14–42 dpi, plants of all treatments showed a decrease of leaf area due to the onset and progression of the infection, and extreme temperature conditions. The summer heat (maximum air temperatures in the greenhouse of 43–47°C for 3 d) reduced leaf growth for all plants in all treatments. After greenhouse shading and readjustment of water supply, the non-inoculated plants increased leaf area during 42–89 dpi, except for the HS genotype that started to increase leaf area after 56 dpi. After an increase in leaf area during the same period, the inoculated HS plants showed a reduced leaf area following the second inoculation that progressively increased disease severity. Finally, at 89 dpi, both genotypes showed a significantly reduced leaf area due to fungal infection (*t*-test, *P*<0.05; Fig. 2C), whereas the LS plants showed a larger leaf areas than the HS plants. The LS genotype showed a 37% larger leaf area of non-inoculated plants and a significantly 42% larger leaf area of inoculated plants compared with the HS genotype. The average leaf area (during –14 to 89 dpi) was similar for both genotypes, whereby the LS plants displayed an increased average leaf area of ~100cm^2^ (Table 1).

**Table 1.** Leaf area (LA; cm^2^) and relative growth rate (RGR; % d^–1^) within 14 d after inoculation, and fresh weight (FW; g) of taproots per treatment of the HS and LS genotype In experiment 1, the LS and HS genotypes were compared. In experiment 2, the HS genotype was measured. Significant differences in taproot fresh weight of plants were measured between the inoculated (+ *C.b.*) and the non-inoculated (– *C.b.*) treatment (*t*-test, *P*<0.05, indicated by an asterisk).

Treatment	Experiment 1		Experiment 2		
	Leaf area (cm^2^)		RGR of taproots (% d^–1^)		FW of taproots (g)
	LS	HS	HS		
			First inoculation, at 0 dpi	Second inoculation, at 56 dpi	
+ *C.b.*	469±30	374±23	6.05±0.31	2.53±0.27	69.7±4.8*
– *C.b.*	476±40	374±24	6.79±0.41	2.90±0.28	97.8±8.7

Arithmetic means ±SE; *n*_LA_=50, *n*_RGR_=4, *n*_FW_=4.

### Disease effects on the taproot of genotypes with contrasting susceptibility

During foliar disease progression, using MRI, it was found that taproot development was reduced in the inoculated treatments. Independent from disease infection, the HS genotype had larger and faster growing taproots than the LS genotype. Throughout experiment 1 during –14 to 89 dpi, the two genotypes differed in increase in taproot volume. Upon *Cercospora* infection, volumetric taproot development was slowed down in both genotypes, whereas no significant difference was found between the inoculated and non-inoculated plants (Fig. 2D). For plants of both treatments, the HS plants had a >2-fold larger taproot volume compared with that of the LS plants at 89 dpi (one-way ANOVA, *P*<0.05, Fig. 2D). The taproot volume correlated with the harvested taproot fresh weight for both genotypes (*R* ^2^=0.994). When inoculated, the HS genotype had a 14% lower taproot volume and 17% lower fresh weight, while the LS genotype had a 30% reduced taproot volume and 21% reduced fresh weight (Fig. 2D,E), compared with the non-inoculated plants.

**Fig. 3.**
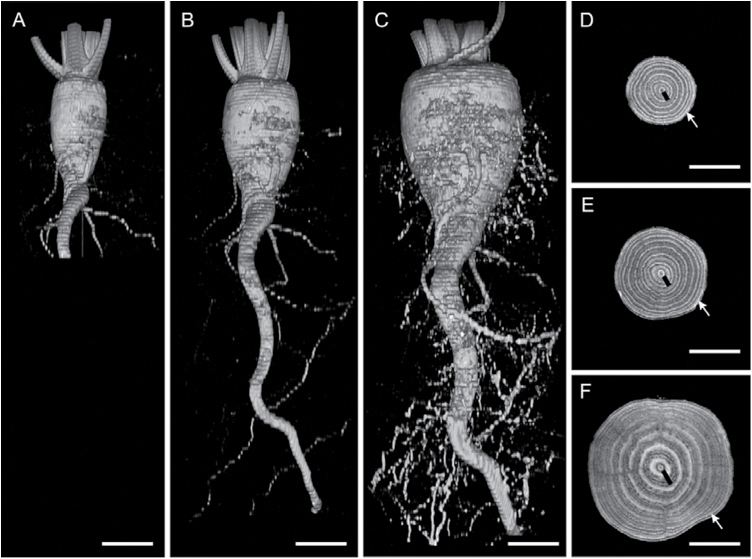
MRI images of a HS plant. The taproot of the sugar beet is shown in isosurfaces and cross-sections at 11 weeks (A, D), 17 weeks (B, E), and 25 weeks after sowing (C, F). (D, E, F) In the cross-sections, the thickness of the innermost cambial ring (no. 1) is marked with a black line; the outermost one with a white arrow. The scale of the isosurfaces and cross-sections is indicated by the white line (2cm). Taproots grew out of the field of view (70×70mm^2^) 16 weeks after sowing; therefore, two measurements per taproot, one set above the other, had to be conducted to capture the root system (B, C). The two MRI projections were concatenated together by image analysis.

The increase in taproot volume was due to beet-specific secondary thickening (Fig. 3A–C). In addition to taproot volume measurements, the development of the width of cambial rings was also measured from the MRI images (Fig. 3D–F). In these cross-sections, darker zones represented the xylem and phloem separated by a narrow bright ring, the cambium. The broader, light grey rings in between were identified as sugar-storing parenchyma, as described in Metzner *et al.* (2014). The thickness of a cambial ring consisted of the summed width of the respective cambium, xylem, phloem, and storage parenchyma.

**Fig. 4.**
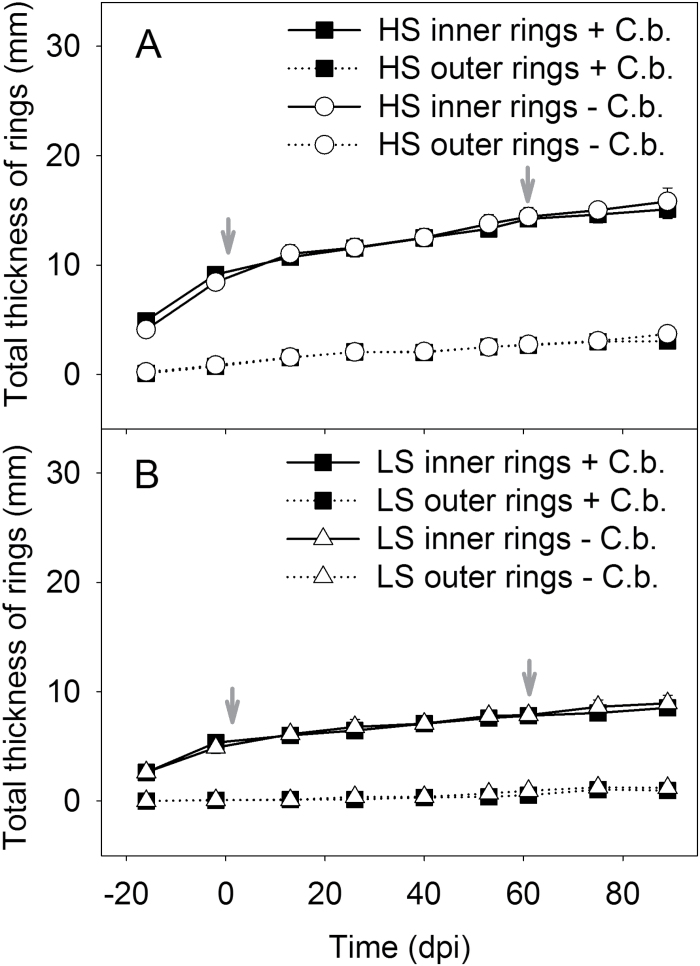
Summed thickness of the inner (1–4) and outer (5–8) cambial rings of the HS (A) and LS (B) genotypes with inoculated (+ *C.b.*) and non-inoculated (– *C.b.*) plants (mean ±SE, *n*=5 plants per treatment) between –14 dpi and 90 dpi, non-invasively measured with MRI. The significant differences between inner and outer cambial rings in the treatments of each genotype during –14 to 89 dpi were analysed by two-way ANOVA, *P*<0.001. The two dates of inoculation with *C. beticola* are indicated by grey arrows.

The observable increase of taproot width was higher in HS plants that had ~2-fold thicker cambial rings throughout the whole experiment during –14 dpi to 89 dpi compared with the LS plants. The increase in taproot width started with the inner cambial rings (rings 1–4) followed by the outer rings (rings 5–8). In both genotypes, the inner cambial rings were significantly larger than the outer rings, whereby the width development of cambial rings was similar (two-way ANOVA, *P*<0.001; Fig. 4). Upon *Cercospora* infection, no difference between inoculated and non-inoculated plants was found (Fig. 4).

### Morphological study of disease effects on the taproot development

The extreme summer temperatures during cultivation led to leaf area loss of plants within all treatments. It was assumed that leaf area loss of the non-inoculated plants was the reason why the differences were small when comparing treatments concerning taproot volume, fresh weight, and ring width development. To confirm the results on pathogen effects observed in experiment 1, and to clarify whether temperature-induced foliar damage affected the root system, a second experiment was conducted under moderate light conditions and with adapted water supply to improve growth conditions compared with experiment 1. In this experiment, the HS genotype was used to confirm the above results and increase the accuracy of measurements concerning changes in the width development of cambial rings.

**Fig. 5.**
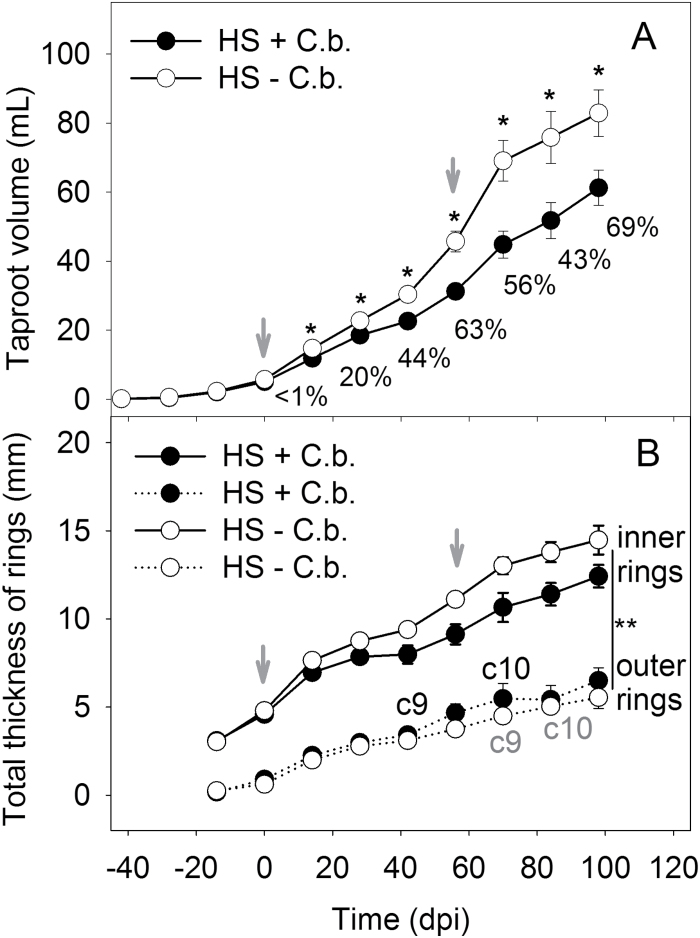
Root development, measured with MRI, and disease severity of the HS genotype scored visually. (A) Taproot volume (ml) and percentage disease severity at the applicable measurement point with increasing average number of infected leaves per plant (ILP_a_), 5–19 leaves; asterisks indicate statistically significant differences between plants of the inoculated (+ *C.b.*) and non-inoculated (– *C.b.*) treatments estimated by a *t*-test, *P*<0.05. (B) Summed thickness of inner (1–4) and outer (5–10) cambial rings between inoculated (+ *C.b.*) and non-inoculated (– *C.b.*) treatment. The appearance of cambial rings 9 (c9) and 10 (c10) was delayed in the non-inoculated plants (grey, non-inoculated; black, inoculated). Differences in cambial rings between genotypes were analysed at 98 dpi by one-way ANOVA, *P*<0.001. Mean values (±SE, *n*=4 per treatment) were calculated during –14 dpi to 98 dpi (A, B). The two dates of inoculation are indicated by grey arrows.

The inoculated HS plants showed a significantly reduced taproot volume already at 14 dpi compared with the non-inoculated plants (Fig. 5A; *t*-test with *P*<0.05), even though the disease severity was low, with only a few leaf spots (<1% infected leaf area; ILP_a_=5). The second inoculation, 56 d after the first one, resulted in a further reduction of the volumetric taproot growth, with a terminal disease severity of 69% (ILP_a_=19) at 97 dpi which increased the volumetric taproot difference between treatments (*t*-test with *P*<0.05; Fig. 5A). To quantify growth reduction, the RGRs of taproots were compared within 14 d after inoculation. The inoculated plants showed a reduced RGR of 11% (first inoculation) and 13% (second inoculation) compared with the non-inoculated plants (Table 1). At 98 dpi, the inoculated plants had a 28% lower taproot volume as determined by MRI (Fig. 5A) and 26% lower taproot fresh weight that was found gravimetrically (Table 1).

An analysis of the width development of cambial rings showed that there was a reduction of the inner rings of the inoculated HS plants during 14–98 dpi compared with the non-inoculated plants (Fig. 5B). After 56 dpi until 89 dpi, the inoculated plants displayed a slightly increased width development of outer rings. Furthermore, there was an earlier width growth of outer cambial rings in the inoculated plants. The outer cambial rings 9 and 10 of the inoculated plants grew 4 and 2 weeks, respectively, before the rings of the non-inoculated plants (Fig. 5B). At 98 dpi, the inner cambial rings were significantly larger than the outer rings (one-way ANOVA with *P*<0.001; Fig. 5B), whereby no significant difference was found upon *Cercospora* infection.

## Discussion

The effects of *Cercospora* leaf spot on sugar beet have been studied in several cases to link leaf damage with losses in sugar yield, and with the defence ability of different sugar beet cultivars (Shane and Teng, 1992; Saito, 1966; Wolf *et al.*, 1998). This includes studies on visual disease scoring (Shane and Teng 1992; Rossi *et al.*, 1999) and hyperspectral disease estimations (Mahlein *et al.*, 2013; Jansen *et al.*, 2014b), as well as molecular analysis of defence gene expression changes after *Cercospora* infection (Weltmeier *et al.*, 2011). Developmental changes in taproot morphology, sugar accumulation, and disease-affected yield losses are usually based on taproots that were dug up at selected time points (Terry, 1968; Shane and Teng, 1992; Vereijssen *et al.*, 2003; Bellin 2007; Trebbi *et al.*, 2009). Investigating plant morphology by harvesting makes it difficult to follow temporal changes in development and to identify small effects at the beginning of biotic stress applications. In this respect, less is known about *Cercospora* effects on the root system of sugar beets at disease onset and how the internal taproot morphology changes during foliar disease progression. Recently, the first non-destructive study of plant–pathogen interaction was carried out that focused on below-ground symptoms of cyst nematode infestation (*Heterodera schachtii*) on the sugar beet root system by using MRI (Hillnhütter *et al.*, 2011). The present non-invasive MRI study of sugar beets grown in soil revealed taproot growth reduction and morphological changes at the beginning of the foliar *Cercospora* infestation while showing low levels of disease severity (<7% infected leaf area). Measurements over several weeks revealed *Cercospora*-induced changes in the development of taproot volume and width of cambial rings.

Irrespective of the resistance level, both genotypes with high (HS) and low susceptibility (LS) showed a reduction in taproot volume already within 14 d after foliar inoculation with *C. beticola*, at a time when only a few leaf spots were observed. It is hypothesized that this diminished root growth might have been caused by the onset of extensive fungal colonization and subsequent expansion in the intercellular space of leaves which was described by Schmidt *et al.* (2008). Although only little macroscopic necrosis was observed at this disease stage, it is likely that many cells were already damaged (Steinkamp *et al.*, 1979) and photosynthetic products as well as nutrients were consumed by fungi growing biotrophically throughout the leaf tissue. This fungal spreading in leaf tissues can be limited by plants showing specific resistance (Feindt *et al.*, 1981). An example of a resistance mechanism is the plant’s ability to reduce the growth of germ tubes towards stomata by suppressing the hydrotropism stimulus (Solel and Minz, 1971). It is also likely that increased defence gene expression can limit fungal spreading (Weltmeier et al., 2011). In a parallel experiment, it was found that LS plants had lower amounts of fungal mycelium in the leaf tissue than HS plants based on the analysis of the fungal calmodulin gene via quantitative real-time PCR (Supplementary Fig. S1 available at *JXB* online). There is increasing evidence that pathogens not only induce direct defence responses, but also alter primary carbohydrate metabolism (Chou *et al.*, 2000; Berger et al., 2004, 2007; Swarbrick *et al.*, 2006; Essmann *et al.*, 2008; Kocal *et al.*, 2008; Bonfig *et al.*, 2010). Based on the sugar degradation of source leaves and the changes in source–sink metabolism, the carbon transport to sink organs such as roots is reduced (Bonfig *et al.*, 2010; Van As and van Duynhoven, 2013). Such a change in carbon transport might be considered to be another factor of the observed taproot growth reduction at disease onset. According to CLS infection, the fresh weight ratio between the taproot and shoot of the HS plants was reduced in experiments 1 and 2 (Table 2). In combination with temperature-induced damage, the taproot–shoot ratio was even more reduced, indicating a complex source–sink relationship between the storage organ and biotically or abiotically stressed leaves. Lower disease severity and larger leaf area in the LS genotype (Table 1; Fig. 2B, C) further indicates an increased investment of carbon and defence at the leaf level. Proportionally, fewer resources could be allocated to the taproots, leading to a significant growth reduction and anatomical changes compared with the HS plants It has been shown frequently that fungi cause yield losses of their host crops by reducing photosynthesis within the infected leaves (Berger *et al.*, 2004; Swarbrick *et al.*, 2006). Also *Cercospora* infection was shown to decrease the photosynthetic efficiency of young sugar beets (Levall and Bornman, 2000) and alter expression of particular genes (Weltmeier *et al.*, 2011). At 16 dpi, leaves with a 3–6% infected area showed significantly reduced chlorophyll fluorescence of the maximum quantum yield (*F*_v_/*F*_m_) investigated by pulse amplitude modulation (PAM) measurements (Levall and Bornman, 2000). Using monitoring PAM fluorometers, it could not be verified here that disease severity of <10% of the measurement area had a clear impact on the photosynthetic efficiency (*F*_v_/*F*_m_) of dark-adapted leaves of a HS genotype (Supplementary Fig. S2). Compared with Levall and Bornman (2000) who measured leaves after 30min dark adaptation, here measurements were conducted on plants during the night (00:00h to 01:00h) after leaves were dark-adapted for 1h. This might lead to lower photosynthetic efficiencies of leaves that were severely infected. These results suggest that diminished root development at the very beginning of leaf infestation can occur at the stage of leaf colonization and tissue invasion without obvious effects on photosynthetic capacity. Therefore, in accordance with several studies, it may be assumed that this effect on taproot growth at an early infestation stage is also caused by the induction of metabolic changes resulting in withdrawal of carbohydrates for storage in taproots.

**Table 2.** Taproot–shoot ratio of the HS and LS genotype in experiments 1 and 2 Ratios were calculated by dividing the fresh weight of the inoculated (+ *C.b.*) by that of the non-inoculated (– *C.b.*) treatment. Variations were determined by calculating the propagation of error according to Barlow (1989).

Treatment	Genotype	Taproot–shoot ratio	
		Experiment 1	Experiment 2
+ *C.b.*	HS	1.3±0.17	1.6±0.14
– *C.b.*	HS	1.4±0.17	1.8±0.23
+ *C.b.*	LS	0.4±0.05	
– *C.b.*	LS	0.4±0.10	

Following the early *Cercospora*-induced effect on taproot growth at an early low disease stage, further losses of taproot biomass triggered by progressing leaf destruction were analysed. In contrast to the HS genotype, the LS plants had a delayed and less severe progression of leaf damage, reflecting both a lower disease severity and a lower number of symptomatic leaves. Comparing the growth pattern of both genotypes, the LS plants showed significantly lower taproot growth in the inoculated and non-inoculated treatment during the whole experiment and lower fresh weight at harvest 90 dpi (Fig. 2D, E). In a parallel experiment, the inoculated and non-inoculated LS plants reached a low fresh weight at 35 dpi, but showed an increased relative sucrose content compared with the HS plants (Supplementary Fig. S3 at *JXB* online). These genotypic contrasts are in good agreement with several studies (Feindt et al, 1981; Shane and Teng, 1992; Weltmeier *et al.*, 2011); regarding this, Smith and Campbell (1996) reported a negative correlation between disease resistance and sucrose yield that included a higher productivity of HS plants under low infection pressure. The present findings indicate a relatively low disease pressure under experimental greenhouse conditions because the LS genotype could not benefit from its genetic background. Disease incidence and severity as well as conidia production are reduced in LS plants (Rossi *et al.*, 1999). However, an ~33% higher sucrose content in LS taproots that was quantified in a preliminary analysis could hardly compensate for a fresh weight that was twice as low as in the HS plants (Supplementary Fig. S3). Defence activation may have a high energy cost and may conflict either with taproot biomass or sugar storage. The expression of defence genes that act directly against pathogens or improve signal perception may compete with the expression of genes that regulate carbon metabolism (Berger *et al.*, 2004; Weltmeier *et al.*, 2011). It would be expected that the genetic advantage of LS plants becomes beneficial at a later disease stage under field conditions that offer optimal requirements for repeatable fungal infestation by frequent conidia release on an increasingly larger number of leaves. In the field, the initial time point of severe disease progression is reached after row closure, which triggers an increase of air humidity and leaf wetting, conditions that are favourable for an abundant production of conidia (Wolf et al., 2001a, b).

In addition to a diminished taproot volume, changes in the morphology of cambial rings were also investigated. As well as a smaller taproot, the LS genotype also had a reduced average width of cambial rings compared with those of the HS genotype (Fig. 4). The inner cambial rings of the LS genotype were almost 2-fold and the outer rings 3-fold thinner, where both genotypes were at a similar developmental stage with eight mature leaves at the time point of the first inoculation. It was found that inner cambial rings were significantly thicker than outer cambial rings (Fig. 4). This can be ascribed to the secondary thickening of the taproot that starts with the storage of sugars in the inner cambial rings followed by the outer cambial rings (Artschwager, 1926). Due to the fungal infection of leaves, a reduction in the average width of inner cambial rings was found. These responses indicate that inner cambial rings were supported by leaves that could only supply a low amount of resources (e.g. sugars) due to pathogen colonization. In contrast, a slightly increased thickness of outer cambial rings was measured, indicating that these were provided with higher amounts of resources by leaves displaying an increased metabolic activity. Furthermore, an earlier change in the width of rings 9 and 10 was found that may be the indirect cause of a fungal infection. These rings grew 2 weeks prior to those of the non-inoculated plants (Fig. 5). This result indicates compensatory effects to CLS-caused growth reduction of the inner cambial rings by an increase in growth of outer cambial rings. The findings point to a primary vascular connection of infected leaves (the first six mature leaves) to the inner four cambial rings. The vascular connection of the first four leaves to the inner two cambial rings was proposed by Zamski and Azenkot (1981). The present observation of leaf-dependent ring growth is in agreement with the study of sectorial sugar accumulation in taproots using a short-lived ^11^CO_2_ tracer for MRI and positron emission tomography (PET) co-localization (Jahnke *et al.*, 2009). In the combined study, sugar beet leaves were exposed to ^11^CO_2_, revealing photoassimilate transport along transport routes to vascular rings in different taproot regions. Hence, the effects on taproot anatomy and growth, such as volume, cambial ring thickness, ring number, and the respective growth rates, may contribute to link disease effects at the canopy level to growth and physiology of the taproot.

Monitoring taproots via MRI allows plant development to be followed with the aim of intervening at specific growth stages; for example, inner and outer cambial ring tissue can be sampled at the onset of cambial width growth to investigate gene expression and metabolic changes. In comparative studies, growing zones of *Arabidopsis* roots, populus, and maize leaves were chosen by microscopic analysis and digital imaging prior to the analysis of molecular and metabolic changes in development (Birnbaum *et al.*, 2003; Matsubara *et al.*, 2006; Rymen *et al.*, 2007). The analysis of genotypes with contrasting levels of disease susceptibility, such as moderately resistant genotypes, might provide further information about disease effects on sugar beet morphology. Morphological traits may be used to characterize yield potential and stress tolerance of genotypes, analysing for example the taproot diameter and the overall proportion of different root parts such as hypocotyl, taproot body, or taproot tail. Morphological taproot imaging could also be combined with chemical shift imaging (CSI) to estimate the current sugar content non-invasively and achieve a more detailed insight into metabolic changes. Thus, resistance-related root traits might be used as an early pre-selection parameter, for example for the detailed characterization of promising parents for breeding programmes, and may elucidate vulnerable plant stages for plant protection at which preventive disease control could be needed to maximize yield gain.

## Supplementary data

Supplementary data are available at *JXB* online.

Figure S1. Fungal growth in the tissue of sugar beet leaves was quantified by molecular analysis.

Figure S2. Photosynthetic efficiency of sugar beet leaves was monitored by chlorophyll fluorometer measurements during disease progression.

Figure S3. Relative sucrose content and fresh weight of taproots, and foliar disease severity quantified in the HS and LS genotypes.

Supplementary Data
