# Supplementary material for: Magnetic resonance imaging of sugar beet taproots in soil reveals growth reduction and morphological changes during foliar Cercospora beticola infestation
Source: J Exp Bot. 2015 Apr 1;66(18):5543–53. doi: 10.1093/jxb/erv109 (PMC4585413; doi:10.1093/jxb/erv109)
Supplement: Supplementary Data [file supp_66_18_5543__index.html]

Magnetic resonance imaging of sugar beet taproots in soil reveals growth reduction and morphological changes during foliar Cercospora beticola infestation — Magnetic resonance imaging of sugar beet taproots in soil reveals growth reduction and morphological changes during foliar Cercospora beticola infestation — Supplementary Data 

# Magnetic resonance imaging of sugar beet taproots in soil reveals growth reduction and morphological changes during foliar *Cercospora beticola* infestation

## Supplementary Data

Data files

**Files in this Data Supplement:**

- Supplementary Data - Supplementary Data
