# Supplementary material for: Magnetic resonance imaging of sugar beet taproots in soil reveals growth reduction and morphological changes during foliar Cercospora beticola infestation
Source: J Exp Bot. 2015 Apr 1;66(18):5543–53. doi: 10.1093/jxb/erv109 (PMC4585413; doi:10.1093/jxb/erv109)
Supplement: Supplementary Data [file supp_erv109_jexbot141341_file001.pdf]

Journal of Experimental Botany

Title: Magnetic Resonance Imaging of sugar beet taproots in soil reveals growth reduction and morphological changes during foliar *Cercospora beticola* infestation

Authors: Schmittgen, Simone; Metzner, Ralf; Van Dusschoten, Dagmar; Jansen, Marcus; Fiorani, Fabio; Jahnke, Siegfried; Rascher, Uwe; Schurr, Ulrich

Supplementary material

Figure S1: Fungal amount in leaves of the HS and LS genotype. The fungal amounts (pg_{DNA} $mg_{leaf\ FW-1}$) were detected by quantitative real-time PCR quantifying the *C. beticola* calmodulin gene (EMBL-EBI, DQ026493). The LS genotype showed an increased and the HS genotype showed a significantly increased fungal amount from 3 to 10 dpi (Mean ±SE) estimated by One-Way ANOVA, Holm-Sidak, P<0.05. Leaves of both genotypes had no leaf symptoms at 3 and 6 dpi and showed first leaf spots at 10 dpi revealing disease severities between 0.2 to 0.5%. Leaves (n = 5 per genotype) were harvested, frozen in liquid nitrogen, grounded and DNA was extracted of about 100 mg using the innuPEP Plant DNA kit (Analytik Jena AG, Jena, Germany). A 229 bp fragment was amplified using the primers (‘5 GCC TTC TCT CTC TTC GTA CGT ACA G ’3/ 5’ GCG AAT GTA CTG AAC TAA CC T CGA CC’3; obtained from Eurofins MWG Operon, Ebersberg, Germany) in a three step PCR (denaturation at 94°C, annealing at 65°C and elongation at 72°C). Fragments were dyed with SYBR Green (Qiagen, Hilden, Germany) and quantified by an optical single-color real-time PCR detections system (Bio-Rad Laboratories GmbH, Munich, Germany). Based on a calibration curve of serial diluted fungal DNA concentrations, the fungal amount in leaf samples was calculated.

Figure S2: Photosynthetic efficiency of a HS breeding line by chlorophyll fluorometer measurements. (A) Leaves were fixed in the leaf clips with an area of 2.2 x 3 cm^2 and measured with Monitoring PAM fluorometers (Heinz Walz GmbH, Effeltrich, Germany) at 10-minute intervals in the course of the day under greenhouse conditions from -3 to 24 days after Cercospora inoculation. To mark the measured leaf area before the inoculation, a rough black outline was drawn by hand. The non-inoculated leaf showed no disease severity (A,B), whereas the inoculated leaves showed a <1% (C), 3% (D), 10% (E) and 60% (F) infected area at 24 dpi of the leaf clip. Leaves are shown with fixed (A) and without leaf clips (C-F). (G) The maximum quantum yield of photosystem (Fv/Fm) of dark-adapted inoculated (+ *C.b.*) and non-inoculated (- *C.b.*) sugar beet leaves was measured every 10 minutes after applying a saturating flash of 3500 µmol m-2 s-1 during the night (from 00.00 h to 01.00 h). At 24 dpi, differences in Fv/Fm between leaves of variable disease severity were measured (leaf #1 with <1%, leaf #2 with 3%, leaf #3 with 10% and #4 with 60%). Mean ±SD (n=4 leaves per treatment).

Figure S3: Relative sucrose content (%) and fresh weight (g_{FW}) of taproots, and foliar disease severity of the HS and LS genotype. (A) Harvested fresh weights of taproots (35 dpi) are given in values (mean ±SE) besides the bars of corresponding relative sucrose content. Relative sucrose content (mean ±SE) of taproots was analyzed by a coupled enzyme assay (Jones et al., 1977) using a micro plate spectrophotometer (Synergy 2 BioTek Instruments Winooski, VT,USA) according to Walter et al. (2002). One quarter per taproot was taken from inoculated (+ *C.b.*) and non-inoculated (-*C.b.*) plants at 35 dpi or 85 das (n=5 + *C.b* plants; n = 3 (HS)/ n=2 (LS) - *C.b.* plants). Frozen samples were grounded in a ball mill (Type MM200, RETSCH, Haan, Germany) and an aliquot of the grounded material (~200 mg) was then analyzed enzymatically. To calculate the relative sucrose content of taproots (%), the absolute sucrose amount (µmol g_{FW}^{-1}) was correlated with the molar mass of sucrose and the sample amount (mg_{FW}). The LS genotype showed higher relative sucrose contents compared with the HS genotype. The significant difference in the -*C.b.* treatment between the HS and LS plants was estimated by t-test, P<0.05. (B) Disease severity progression (mean ±SE) with increasing average number of infected leaves per plant (ILP_a), 5 - 30 leaves for LS and 6 - 20 leaves for HS from 7 to 35 dpi. Plants were inoculated with a conidia-water solution of 6 x 10^4 conidia mL^{-1}. Plants were grown under greenhouse conditions in 1L soil-filled pots.

Supplementary data

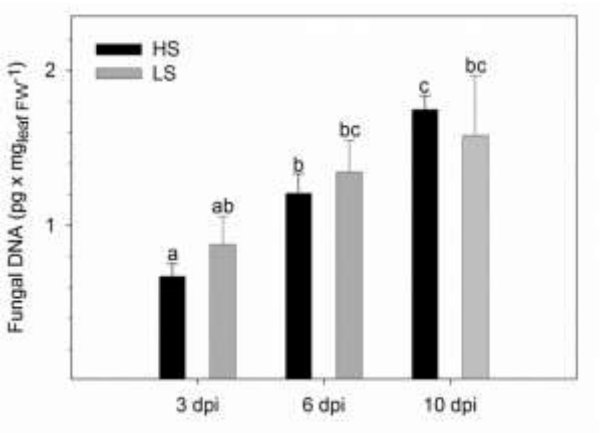

Figure S1

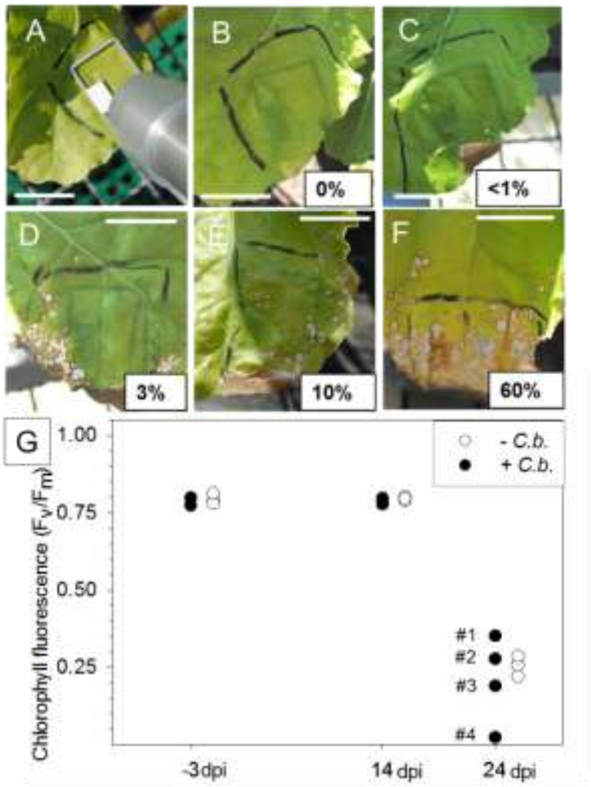

Figure S2

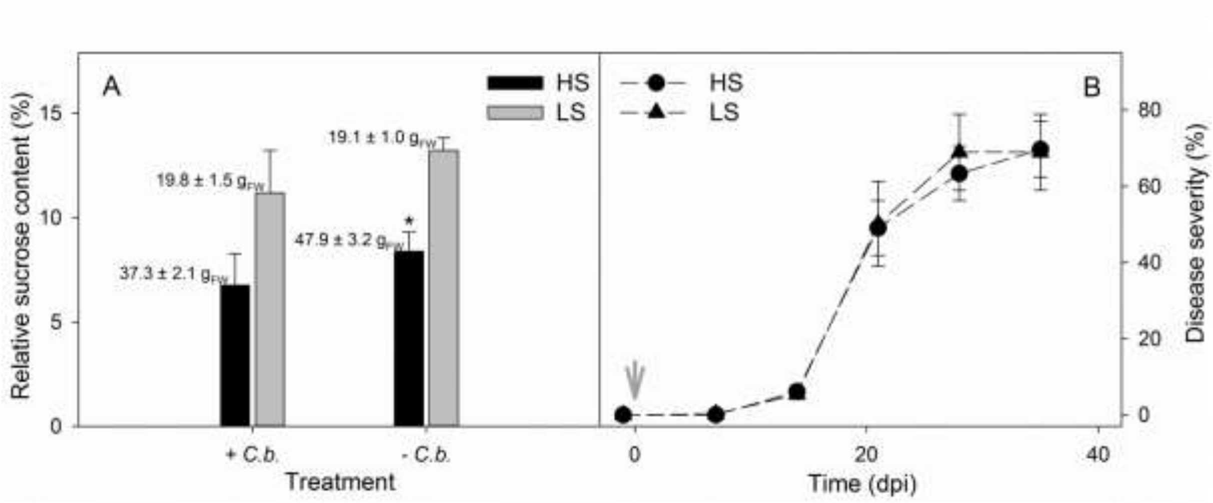
A
B
HS
LS
HS
LS
Relative sucrose content (%)
Disease severity (%)
37.3 ± 2.1 gFW
19.8 ± 1.5 gFW
47.9 ± 3.2 gFW
19.1 ± 1.0 gFW
*
0
5
10
15
0
20
40
60
80
+ C.b.
- C.b.
Treatment
0
20
40
Time (dpi)

Figure S3
